# Preferred and Actual Location of Death in Adolescents and Young Adults With Cancer

**DOI:** 10.1001/jamanetworkopen.2024.54000

**Published:** 2025-01-14

**Authors:** Oreofe O. Odejide, Colin Cernik, Hajime Uno, Lauren Fisher, Lanfang Xu, Cecile A. Laurent, Nancy Cannizzaro, Julie Munneke, Robert M. Cooper, Joshua R. Lakin, Corey M. Schwartz, Mallory Casperson, Andrea Altschuler, Lori Wiener, Lawrence Kushi, Chun R. Chao, Jennifer W. Mack

**Affiliations:** 1 Division of Population Sciences, Dana-Farber Cancer Institute, Boston, Massachusetts; 2 Department of Medical Oncology, Dana-Farber Cancer Institute, Boston, Massachusetts; 3 MedHealth Analytics Inc, Sugar Land, Texas; 4 Division of Research, Kaiser Permanente Northern California, Oakland; 5 Department of Research and Evaluation, Kaiser Permanente Southern California, Pasadena; 6 Department of Pediatric Oncology, Kaiser Permanente Southern California, Pasadena; 7 Department of Psychosocial Oncology and Palliative Care, Dana-Farber Cancer Institute, Boston, Massachusetts; 8 Division of Medical Oncology, Kaiser Permanente Northern California, Oakland; 9 Cactus Cancer Society, Oakland, California; 10 Psychosocial Support and Research Program, National Cancer Institute, Bethesda, Maryland; 11 Department of Pediatric Oncology, Dana-Farber Cancer Institute, Boston, Massachusetts

## Abstract

**Importance:**

Adolescent and young adult (AYA) patients with advanced cancer often die in hospital settings. Data characterizing the degree to which this pattern of care is concordant with patient goals are sparse.

**Objective:**

To evaluate the extent of concordance between the preferred and actual location of death among AYA patients with cancer.

**Design, Setting, and Participants:**

This multicenter retrospective cohort study included AYA patients (aged 12-39 years) with cancer who died between January 1, 2003, and December 31, 2019, after receiving care at Dana-Farber Cancer Institute and Kaiser Permanente Northern California or who died between January 1, 2009, and December 31, 2019, after receiving care at Kaiser Permanente Southern California. Data were analyzed from January 12 to July 1, 2024.

**Exposure:**

Death due to cancer.

**Main Outcomes and Measures:**

Medical record documentation of discussions about preferred location of death, actual location of death, and concordance between preferred and actual location of death.

**Results:**

The analytic population included 1929 AYA decedents, of whom 1049 (54.4%) were female; 227 (11.8%), Asian; 157 (8.1%), Black; 514 (26.6%), Hispanic; and 1184 (61.4%), White. Median age at death was 32 (IQR, 25-37) years. A total of 1226 AYA patients (63.6%) had a documented discussion about preferred location of death. Among those with a documented discussion, 594 (48.5%) did not have a documented preference, 402 (32.8%) wanted to die at home, 177 (14.4%) preferred a hospital death, and 48 (3.9%) desired inpatient hospice. Eight hundred and thirty patients (43.0%) died in acute care settings (256 [13.3%] intensive care unit [ICU], 548 [28.4%] hospital [non-ICU], and 26 [1.3%] emergency department), while 643 (33.3%) died at home and 47 (2.4%) in an inpatient hospice. Among the 528 patients with both a documented preferred death location of home, hospital, or inpatient hospice and documented death in one of these locations, the concordance between preferred and actual location of death was 401 (75.9%). One hundred and sixty-four of 172 patients (95.3%) who preferred a hospital death died there; 224 of 317 (70.7%) who preferred a home death died at home, and 13 of 39 (33.3%) who desired to die in inpatient hospice did so.

**Conclusions and Relevance:**

Although many AYA patients with cancer died in their preferred location, over one-quarter of those who desired to die at home did not realize this goal. These findings highlight the need for effective solutions to enable goal-concordant care for this population.

## Key Points

**Question:**

What is the concordance between preferred and actual location of death for adolescent and young adult (AYA) patients with cancer?

**Findings:**

In this cohort study of 1929 AYA decedents, 1226 (63.6%) had a documented discussion about their preferred location of death, with home being most frequently desired. Among these, 224 of 317 (70.7%) who wanted to die at home were able to do so, as were 164 of 172 (95.3%) who preferred a hospital death.

**Meaning:**

The fact that over a quarter of AYA patients with cancer who preferred to die at home were unable to do so suggests a need for effective interventions to improve goal-concordant end-of-life care for AYA patients with cancer.

## Teaser

This cohort study assesses how often young adults and adolescents with cancer are engaged in discussion about their preferred location of death and whether the actual location was concordant with their preferred location.

## Introduction

Location of death is an important factor in assessing the quality of end-of-life (EOL) care for adolescents and young adult (AYA) patients with cancer. Most of the 9000 AYA patients who die of cancer each year in the US do so in acute care settings,^1,2,3,4,5,6,7,8^ with 1 study reporting that almost 7 of 10 patients died in the hospital.^7^ This pattern of care stands in contrast with the general societal perception that a “good death” is one that occurs at home surrounded by loved ones. While existing high rates of in-hospital deaths for AYA patients with cancer are concerning, lack of data regarding how such care maps onto patient preferences limits a clear understanding of whether these high rates of hospital care represent suboptimal EOL care.

Burgeoning efforts to define AYA patient–specific EOL quality indicators based on input from AYA patients, their caregivers, and their clinicians have identified a greater emphasis on preference-sensitive care in contrast to quality indicators developed for older adults.^9,10,11,12^ Preferred location of death for AYA patients with cancer is highly variable,^13^ and rather than defining hospital death as indicative of poor care regardless of patient preference, it is now recommended to assess EOL care for AYA patients based on whether they had the opportunity to die in their preferred location.^9,10,14,15^ To develop effective interventions to optimize EOL care for AYA patients with cancer, we need comprehensive studies quantifying the extent to which AYA patients experience goal-concordant care with respect to location of death.

While various studies have documented goals of care discussions or location of death for AYA patients with cancer,^1,2,3,7,13,16,17,18,19,20^ research to date examining congruence between preferred and actual locations of death is sparse.^21^ To address this gap in AYA cancer research, we examined preferred and actual location of death among a large cohort of AYA patients who died of cancer. We used medical records to characterize how often AYA patients engaged in discussions regarding their preferred location of death, what their preferences were, and whether these changed over time. Importantly, we aimed to determine how often their actual location of death was concordant with their preferred location. We hypothesized that although most AYA patients would prefer a home death, a considerable proportion would still report a preference for dying in the hospital. We also anticipated that goal-concordant location of death would be highest in instances where hospital death was preferred.

## Methods

### Study Population

In this cohort study, we included AYA patients with cancer (aged 12-39 years) who died after receiving care at a large academic cancer center, Dana-Farber Cancer Institute (DFCI), Boston, Massachusetts, and 2 large integrated health care delivery systems, Kaiser Permanente Northern California (KPNC) in Oakland and Kaiser Permanente Southern California (KPSC) in Pasadena. DFCI and KPNC included patients who died between January 1, 2003, and December 31, 2019. Because of limited resources for abstraction of paper records, KPSC patients who died between January 1, 2009, and December 31, 2019, were included, matching years of available electronic records. While this was a decedent cohort, to ensure that discussions about location of death would have been possible, we focused on patients with anticipated deaths by including patients with stage IV cancer and stages I to III disease with either a new metastasis or more than 1 chemotherapy regimen with more than 90 days between regimens, suggesting cancer recurrence.^22^ Institutional review board approval was obtained at each site. Informed consent was waived because the study included only decedents. This study followed the Strengthening the Reporting of Observational Studies in Epidemiology (STROBE) reporting guideline.

### Data Source

We combined electronic health data and medical records to ascertain data elements for this analysis. For DFCI, an electronic clinical database—the Oncology Data Retrieval System^23^—was queried to identify eligible patients and their sociodemographic and treatment information. These data were complemented by medical record review. For KPNC and KPSC, data for all patients diagnosed with and/or treated for cancer during the study period were obtained from Surveillance, Epidemiology, and End Results–affiliated cancer registries. In addition, we used clinical databases that included membership, diagnosis, procedures, pharmacy, infusions, and outside claims. Full medical records were also accessible electronically for the study period.

We used electronic clinical databases to ascertain study variables such as sociodemographic (eg, race and ethnicity) and clinical characteristics. To determine the presence and content of discussions regarding preferred location of death, trained abstractors (including L.F.) conducted manual medical record review. After an initial period of training and, when possible, dual abstraction, reviewers performed coding independently, with weekly calls across sites to address and resolve discrepancies.

### Study Measures

#### Preferred Location of Death

Documented discussions about preferred location of death were abstracted from the medical records. Preferred location of death was categorized as (1) home, (2) inpatient hospice, (3) hospital, (4) not documented (ie, discussions occurred but did not result in a decision or documentation of preferred location), and (5) other. Because some patients had multiple discussions about preferred location of death and because preferences evolved over time for some patients, each documented discussion on a different calendar day was abstracted. When multiple discussions were found in the medical record, the preferred location noted in the last discussion before death was used to determine goal-concordant care (ie, concordance was achieved if the patient died in their last recorded preferred place).

#### Location of Death

We ascertained patients’ location of death from the medical record and categorized this into (1) home, (2) inpatient hospice, (3) hospital, (4) intensive care unit (ICU), (5) emergency department, (6) other, and (7) not documented. Hospital, ICU, and emergency department were jointly categorized as acute care settings.

### Statistical Analysis

Data on the preferred and actual location of death were generated as percentages. We examined differences in preferred and actual locations of death by age category (12-24 years at death, to match the World Health Organization definition of AYA, vs 25-39 years to match the aspect of the National Cancer Institute definition that does not overlap with the World Health Organization) using Fisher exact or Pearson χ^2^ test. We described the location of death for patients who had a documented discussion about preferred place of death (regardless of whether preference was documented) and those without a documented discussion. Next, we examined the association between the timing of the last documented discussion and location of death. We also assessed the degree of concordance between preferred and actual location of death for home, hospital (non-ICU), and inpatient hospice. We then built a single logistic regression model to assess whether sociodemographic factors (age at death, sex, race, and ethnicity), clinical factors (cancer diagnosis and time from diagnosis to death), year of death, or site of care were associated with attaining concordance between the preferred and actual location of death. Statistical tests were 2 sided, with *P* < .05 used to determine statistical significance. All analyses were performed using R statistical software, version 4.3.2 (R Core Team). Analyses were performed from January 12 to July 1, 2024.

## Results

Of 1929 AYA patients included in this study, 283 (14.7%) had hematologic cancers, 1506 (78.0%) had solid neoplasms, and 140 (7.3%) had unknown or other cancers. Just over half of the population was female (1049 [54.4%] vs 875 [45.4%] male and 5 [0.3%] not documented), and the median age at death was 32 (IQR, 25-37) years. With respect to race, 5 patients (0.3%) were American Indian or Alaska Native; 227 (11.8%), Asian; 157 (8.1%), Black or African American; 14 (0.7%), Native Hawaiian or Other Pacific Islander; 1184 (61.4%), White; 11 (0.6%), multiracial; 38 (2.0%), other (including Asian Indian, Hispanic, Jordanian, Mexican, and declined to state); and 293 (15.2%) not documented. With respect to ethnicity, 514 patients (26.6%) were Hispanic or Latino and 653 (33.9%), not documented. Other cohort characteristics are displayed in Table 1.

**Table 1.** Cohort Characteristics

Characteristic	No. (%) of patients (N = 1929)
Age at death, y	
Mean (SD)	30 (8)
Median (IQR)	32 (25-37)
Age at diagnosis, y	
Mean (SD)	28 (8)
Median (IQR)	30 (23-35)
No. unknown	37
Sex	
Female	1049 (54.4)
Male	875 (45.4)
Not documented	5 (0.3)
Race	
American Indian or Alaska Native	5 (0.3)
Asian	227 (11.8)
Black or African American	157 (8.1)
Native Hawaiian or Other Pacific Islander	14 (0.7)
White	1184 (61.4)
Multiracial	11 (0.6)
Not documented	293 (15.2)
Other^a^	38 (2.0)
Ethnicity	
Hispanic or Latino	514 (26.6)
Not documented	653 (33.9)
Not Hispanic or Latino	762 (39.5)
Primary cancer site	
Bone or soft tissue	264 (13.7)
Brain	170 (8.8)
Breast	265 (13.7)
GI	369 (19.1)
GU	214 (11.1)
Head, neck, thyroid	72 (3.7)
Leukemia	164 (8.5)
Lung	85 (4.4)
Lymphoma	119 (6.2)
Melanoma or skin	67 (3.5)
Other	136 (7.1)
Unknown	4 (0.2)
Stage at diagnosis	
I to III	1119 (58.0)
IV	537 (27.8)
Unknown	273 (14.2)

Abbreviations: GI, gastrointestinal; GU, genitourinary.

^a^ Included Asian Indian, Hispanic, Jordanian, Mexican, and “decline to state.”

### Discussions About Preferred Location of Death

About two-thirds of patients (1226 [63.6%]) had at least 1 documented discussion about preferred location of death, with a median of 15 (IQR, 5-42) days between the first documented discussion and death (Table 2 and eFigure in Supplement 1). Among the patients with a documented discussion, 594 (48.5%) did not have a documented preference regarding location of death. For those with a documented preference, home was the most common (402 [32.8%]), followed by hospital (177 [14.4%]) and inpatient hospice (48 [3.9%]). Younger AYA patients (12-24 years) tended to have a greater preference for hospital death compared to older AYA patients (66 of 307 [21.5%] vs 111 of 919 [12.1%]; *P* < .001) (Table 2).

**Table 2.** Preferences for Location of Death and Actual Location of Death for Adolescents and Young Adults With Cancer

Characteristic	Age group, No. (%) of patients			*P* value^a^
	All (N = 1929)	12-24 y (n = 458)	25-39 y (n = 1471)	
Discussion about preferred location of death (N = 1929)	1226 (63.6)	307 (67.0)	919 (62.5)	.08
Last documented preference for location of death (n = 1226)				
Home	402 (32.8)	101 (32.9)	301 (32.8)	<.001
Hospital	177 (14.4)	66 (21.5)	111 (12.1)	
Inpatient hospice	48 (3.9)	8 (2.6)	40 (4.4)	
Not documented	594 (48.5)	130 (42.3)	464 (50.5)	
Other	5 (0.4)	2 (0.7)	3 (0.3)	
Location of death (n = 1929)				
Emergency department	26 (1.3)	7 (1.5)	19 (1.3)	.002
Home	643 (33.3)	124 (27.1)	519 (35.3)	
Intensive care unit	256 (13.3)	74 (16.2)	182 (12.4)	
Inpatient hospice	47 (2.4)	9 (2.0)	38 (2.6)	
Inpatient hospital, non–intensive care unit	548 (28.4)	140 (30.6)	408 (27.7)	
Not documented	257 (13.3)	54 (11.8)	203 (13.8)	
Other	152 (7.9)	50 (10.9)	102 (6.9)	

^a^ Calculated using Pearson χ^2^ test.

Among the 1226 patients with documented discussions, 246 (20.1%) had more than 1 discussion. Of these, 172 patients (69.9%) stated the same preference at their first and last documented discussion. For the 124 patients whose last recorded preference was home, 102 (82.3%) had the same initial preference. The remaining 22 patients (17.7%) had an initial preference of hospital death (12 [9.7%]) or inpatient hospice (2 [1.6%]) or did not have a documented preference (8 [6.4%]). When hospital was the last documented preferred location of death, 45 patients (62.5%) also chose hospital at the first discussion. The remaining 27 patients (37.5%) initially had a documented preference to die at home (18 [25.0%]) or did not have a documented preference (9 [12.5%]). Among 33 AYA patients without a documented preference at their first discussion, 21 (63.6%) had home, hospital, or inpatient hospice documented by their last discussion.

The timing of the last documented discussion was associated with preferred location of death (Table 3). When the last recorded discussion occurred more than 30 days before death (n = 219), few patients preferred a hospital death (7 [3.2%]), while close to one-third (68 [31.1%]) preferred a home death and 142 (64.8%) did not have a documented preference (*P* < .001). When the last documented discussion occurred within 7 days before death (n = 624), 155 patients (24.8%) preferred a hospital death, 192 (30.8%) chose a home death, and 247 (39.6%) did not have a documented preference (*P* < .001).

**Table 3.** Timing of Conversations About Location of Death and the Association With Preferences or Care Received

Characteristic	Timing of discussion before death, No. (%) of patients			*P* value^a^
	≤7 d (n = 624)	8-30 d (n = 383)	>30 d (n = 219)	
Last documented preference for location of death (n = 1226)				
Home	192 (30.8)	142 (37.1)	68 (31.1)	<.001
Hospital	155 (24.8)	15 (3.9)	7 (3.2)	
Inpatient hospice	27 (4.3)	19 (5.0)	2 (0.9)	
Not documented	247 (39.6)	205 (53.5)	142 (64.8)	
Other	3 (0.5)	2 (0.5)	0	
Location of death (n = 1226)				
Emergency department	7 (1.1)	2 (0.5)	1 (0.5)	<.001
Home	141 (22.6)	186 (48.6)	122 (55.7)	
Intensive care unit	136 (21.8)	12 (3.1)	7 (3.2)	
Inpatient hospice	15 (2.4)	14 (3.7)	7 (3.2)	
Inpatient hospital, non-ICU	259 (41.5)	58 (15.1)	29 (13.2)	
Unknown	29 (4.6)	41 (10.7)	33 (15.1)	
Other	37 (5.9)	70 (18.3)	20 (9.1)	

^a^ Calculated using Fisher exact test.

### Location of Death

In the full cohort (N = 1929), 830 patients (43.0%) died in acute care settings (256 [13.3%] in the ICU, 548 [28.4%] in the hospital [non-ICU], and 26 [1.3%] in the emergency department), while 643 (33.3%) died at home and 47 (2.4%) died in an inpatient hospice. Two hundred and fifty-seven patients (13.3%) did not have a documented location of death in available records. Younger AYA patients (12-24 years) had a lower rate of dying at home compared with older AYA patients (124 of 458 [27.1%] vs 519 of 1471 [35.3%]; *P* = .002) (Table 2).

### Association Between Preferred Location of Death Discussions and Actual Location of Death

When patients had a discussion regarding preferred place of death (even when preferred location was not documented), 449 of 1226 (36.6%) died at home vs 194 of 703 (27.6%) who did not have a discussion (eTable in Supplement 1). The timing of the last recorded discussion about location of death was associated with location of death (Table 3). Individuals with the last documented discussion more than 30 days before death or 8 to 30 days before death had higher rates of dying at home (122 of 219 [55.7%] and 186 of 383 [48.6%], respectively) and lower rates of dying in acute care settings (37 of 219 [16.9%] and 72 of 383 [18.8%], respectively). On the other hand, individuals whose last documented discussion was within 7 days before death had lower rates of home death (141 of 624 [22.6%]) and higher rates of death in acute care settings (402 of 624 [64.4%]).

Among all patients with both a documented preferred location of death for home, hospital, or inpatient hospice and a documented location of death in one of these 3 locations (n = 528), concordance between preferred and actual location of death occurred in 401 (75.9%). Almost all patients who preferred to die in the hospital died there (164 of 172 [95.3%]). In contrast, more than two-thirds of those whose preference was a home death died at home (224 of 317 [70.7%]), and only one-third of patients who desired to die in an inpatient hospice did so (13 of 39 [33.3%]) (Figure). When patients did not die in their preferred place, the most common place of death was the hospital. Sociodemographic factors (age, sex, race, and ethnicity), year of death, cancer type, and care site were not associated with concordance between preferred and actual location of death (Table 4).

**Figure.**
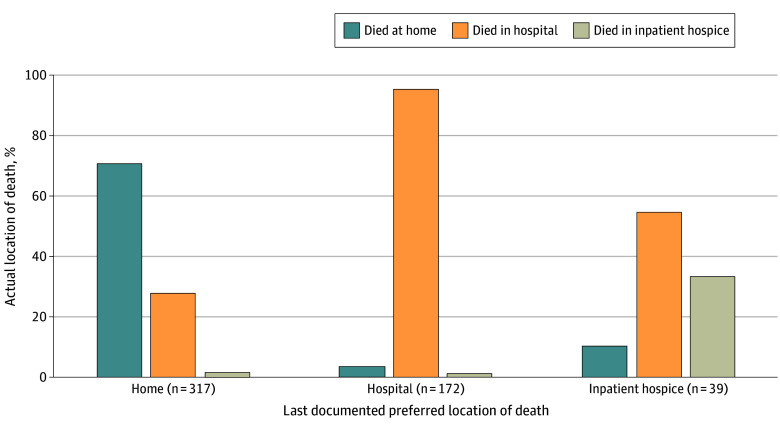
Concordance Between Preferred and Actual Location of Death Includes patients who had a documented preference for location of death that was home, hospital, or inpatient hospice and had a documented actual location of death (n = 528). Overall concordance rate was 401 (224 + 164 + 13) of 528 patients (75.9% [95% CI, 72.3%-79.6%]).

**Table 4.** Odds of Adolescents and Young Adults With Cancer Dying in the Place of Their Choosing

Characteristic	OR (95% CI)^a^	*P* value
Age at death, y		
12-24	1 [Reference]	NA
25-39	0.65 (0.38-1.09)	.11
Year of death	1.00 (0.93-1.07)	.94
Sex		
Female	1 [Reference]	NA
Male	0.96 (0.62-1.49)	.86
Time from diagnosis to death, y	1.02 (0.95-1.11)	.56
Race		
White	1 [Reference]	NA
Asian	0.66 (0.34-1.30)	.22
Black or African American	0.73 (0.34-1.67)	.43
Not documented	0.78 (0.42-1.47)	.43
Ethnicity		
Hispanic or Latino	1 [Reference]	NA
Not documented	1.11 (0.54-2.26)	.78
Not Hispanic or Latino	1.36 (0.72-2.57)	.34
Cancer diagnosis		
Hematologic	1 [Reference]	NA
Brain	1.83 (0.72-5.12)	.22
Solid	0.83 (0.45-1.49)	.55
Site of care		
DFCI	1 [Reference]	NA
KPNC	1.37 (0.75-2.53)	.31
KPSC	1.08 (0.63-1.89)	.77

Abbreviations: DFCI, Dana-Farber Cancer Institute; KPNC, Kaiser Permanente Northern California; KPSC, Kaiser Permanente Southern California; NA, not applicable; OR, odds ratio.

^a^ Model was mutually adjusted for age at death, year of death, sex, time from diagnosis to death, race, ethnicity, cancer diagnosis, and site of care. Includes 507 patients (21 excluded due to missingness), of whom 386 died in the place of their choosing.

## Discussion

In this large multicenter retrospective cohort study of AYA decedents, we found that almost two-thirds of patients had discussions about their preferred location of death. When a preference was expressed, it was most frequently to die at home, and nearly 1 in 6 AYA patients expressed a preference to die in the hospital. Ultimately, about three-quarters of this AYA cohort died in their preferred location, with the highest levels of concordance between preferred and actual location of death occurring among those who wanted to die in hospitals. Although many AYA patients with cancer had discussions about their preferred place of death and most achieved congruence in care, a considerable proportion did not die in their preferred location, and this discordance was more pronounced for AYA patients who wanted to die at home.

Although most patients who expressed a preference for home death died there, nearly one-third ended up dying in acute care settings. On the other hand, almost everyone who opted for a hospital death died there. These findings are consistent with a study of 84 AYA patients in the UK,^21^ in which all patients who preferred to die in the hospital did so and over one-third of those who wanted to die at home died in the hospital. This suggests that, for many, hospital is the default location for death, and patients may face barriers to achieving a home death. Dying at home requires significant support to manage physical and emotional symptoms, and family members often have to take on substantial caregiver duties, including toileting, bathing, administering medications, and even managing tubes and drains. Indeed, caregivers of AYA patients spend on average 32.5 h/wk providing care for their loved ones.^24^ Although hospice provides home-based services to support patients and facilitate a home death when preferred, services are typically intermittent. Thus, family caregivers are responsible for delivering a substantial portion of the required care for their loved ones, which may result in a high physical, emotional, and financial toll.^24,25,26,27^ AYA patients may also have escalating distressing symptoms that are difficult to manage at home, ultimately resulting in hospital admission and hospital death. In addition, AYA patients who desire to die at home may decide to die in the hospital to protect younger siblings or children from the perceived trauma of a home death.^13^

Aligning care with patients’ priorities is a key tenet of high-quality EOL care^28^; therefore, we need solutions to enable AYA patients to die at home when they desire to do so. One potential step would be to provide practical and psychological support for family caregivers. Increasing access to continuous in-home hospice nursing care for AYA patients with escalating physical symptoms has been proposed as an intervention to support goal-congruent location of death for patients who desire to die at home.^29^ Expert psychosocial care for siblings or children of AYA patients with cancer may also bolster mental health support and alleviate worries regarding a home death.

While disparities in EOL care have been described for AYA patients with cancer,^1,2,16,17^ we found no association of race or ethnicity in the attainment of congruence in preferred and actual location of death. Prior work^16^ has found that Black and Hispanic AYA patients with cancer are less likely to have discussions about preferred location of death compared with non-Hispanic White patients. In addition, Asian, Black, and Hispanic AYA patients with cancer are more likely to die in hospital settings compared with non-Hispanic White patients.^1,2,17^ These known differences in rates of discussion and actual location of death did not translate into significant differences in goal-congruent care in our analysis. Similarly, although younger AYA patients tended to have low rates of dying at home, there were no differences in goal-concordant care between younger and older AYA patients. This suggests that perhaps one of the most critical factors in dying where one wishes is to engage in discussions about preferred location of death. When these discussions occur, patients’ race, ethnicity, or age may be less likely to differentially impact alignment of preferences with care.

To ensure that care truly reflects the desires of AYA patients regardless of their racial or ethnic background, engaging in timely discussions about preferred location of death is vital. It is noteworthy that one-third of AYA decedents in this cohort did not have a documented discussion about location of death. Clinicians may hesitate to bring up conversations about preferred location of death because it requires addressing the possibility of death. However, this topic is very important to AYA patients with cancer,^10^ and these discussions are an essential aspect of patient-centered care. To overcome the discomfort of engaging in these discussions, clinicians could conduct these discussions as hypothetical (eg, “Have you thought about where you would most like to receive care if it became clear you were not going to survive?”) rather than definitive. Moreover, even among those who had a documented discussion, the median time from the first discussion to death was only 15 days. While defining “timely” for discussions about location of death is challenging, discussions occurring very close to death likely make it difficult to set up all the necessary resources to facilitate death in patients’ desired locations, especially when the preference is home.

We found that almost half of AYA patients with a discussion did not have a documented preferred location of death. Moreover, preferences changed over time among AYA patients who had more than 1 discussion, with almost two-thirds without a documented preference at their first discussion identifying a specific location at their last discussion. Taken together, these findings suggest that AYA patients may experience ambivalence about preferred location of death, and clarity about specific details may change with time. Accordingly, it is important for clinicians to make space for recurring conversations to accommodate patients’ evolving desires and changing circumstances.^30,31^ In our analysis, 25% of AYA patients who opted for a hospital death at their final discussion had expressed a preference to die at home at their first discussion. This shift may indicate true deep-seated preferences. However, it may also be reflective of patients’ concern that there are inadequate resources to facilitate a peaceful and painless death at home that minimizes burden on family caregivers.^10^ This illustrates the need for clinicians to not only elicit patients’ preferences regarding location of death but to also aim to understand the drivers of those preferences so that appropriate resources can be provided to address underlying concerns.

### Strengths and Limitations

This study has strengths, including a large and diverse cohort of AYA patients and moving beyond assessing intensity of care with respect to location of death to a more complete picture of alignment between preferences and actual care. However, we acknowledge limitations to our study. Although there was detailed medical record abstraction, the observed rate of discussions about preferred location of death may be an underestimate, as not all discussions may be documented.^32^ Second, although we abstracted details and content of each discussion, we cannot ascertain the quality of communication or patient experiences with discussions. Third, we identified AYA decedents retrospectively, but clinicians and patients encounter prognostic uncertainty regarding the timing of death prospectively, which may influence discussions regarding location of death. We attempted to address this limitation by including AYA patients with anticipated deaths, for whom EOL care discussions and planning would be appropriate. Fourth, while we included a diverse population from 3 centers in urban settings, our results may not be generalizable to community centers and rural populations. We also lacked details on socioeconomic variables (eg, income), which may potentially influence location of death or hospice access. Finally, in instances where patients received discordant care, we were not able to identify the factors that resulted in misalignment.

## Conclusions

In this cohort study of 1929 AYA patients who died of cancer, we shed light on the extent to which AYA patients had the opportunity to die in the location of their choosing. Although it is encouraging that many patients died in their preferred location, the fact that nearly 1 in 3 AYA patients who wanted to die at home received discordant care raises concerns regarding the quality of EOL care for this population. Our findings emphasize the need for concerted efforts to help AYA patients with cancer achieve patient-centered EOL care, where the gap between preferred and actual location of death is eliminated.

## Notes

Supplement 1. **eTable.** Location of Death According to the Presence of a Discussion About Preferred Location Among Adolescents and Young Adults With Cancer **eFigure.** Documented Discussions Regarding Preferences for Location of Death by Adolescents and Young Adults With Cancer

Supplement 2.
Data Sharing Statement
