# Supplementary material for: Preferred and Actual Location of Death in Adolescents and Young Adults With Cancer
Source: JAMA Netw Open. 2025 Jan 14;8(1):e2454000. doi: 10.1001/jamanetworkopen.2024.54000 (PMC11733697; doi:10.1001/jamanetworkopen.2024.54000)
Supplement: Supplement 1. — eTable. Location of Death According to the Presence of a Discussion About Preferred Location Among Adolescents and Young Adults With Cancer eFigure. Documented Discussions Regarding Preferences for Location of Death by Adolescents and Young Adults With Cancer [file jamanetwopen-e2454000-s001.pdf]

Supplementary Online Content

Odejide OO, Cernik C, Uno H, et al. Preferred and actual location of death in adolescents and young adults with cancer. *JAMA Netw Open*. 2025;8(1):e2454000. doi:10.1001/jamanetworkopen.2024.54000

eTable. Location of Death According to the Presence of a Discussion About Preferred Location Among Adolescents and Young Adults With Cancer

eFigure. Documented Discussions Regarding Preferences for Location of Death by Adolescents and Young Adults With Cancer

This supplementary material has been provided by the authors to give readers additional information about their work.

© 2025 Odejide OO et al. *JAMA Network Open*.

eTable. Location of Death According to the Presence of a Discussion About Preferred Location Among Adolescents and Young Adults With Cancer

	Patients who had a discussion about preferred location of death (N=1226)		Patients who did not have a discussion about preferred location of death (N=703)
**Location of Death**	**Had a discussion, and preferred location was documented, N = 632 n (%)**	**Had a discussion, but preferred location was not documented N = 594 n (%)**	**n (%)**
Home	235 (37.2)	214 (36.0)	194 (27.6)
Intensive Care Unit	87 (13.8)	68 (11.4)	101 (14.4)
Inpatient hospital, non-intensive care unit	184 (29.1)	162 (27.3)	202 (28.7)
Inpatient Hospice	20 (3.2)	16 (2.7)	11 (1.6)
Emergency Department	5 (0.8)	5 (0.8)	16 (2.3)
Other	39 (6.2)	88 (14.8)	25 (3.6)
Unknown	62 (9.8)	41 (6.9)	154 (21.9)

© 2025 Odejide OO et al. *JAMA Network Open*.

eFigure. Documented Discussions Regarding Preferences for Location of Death by Adolescents and Young Adults With Cancer

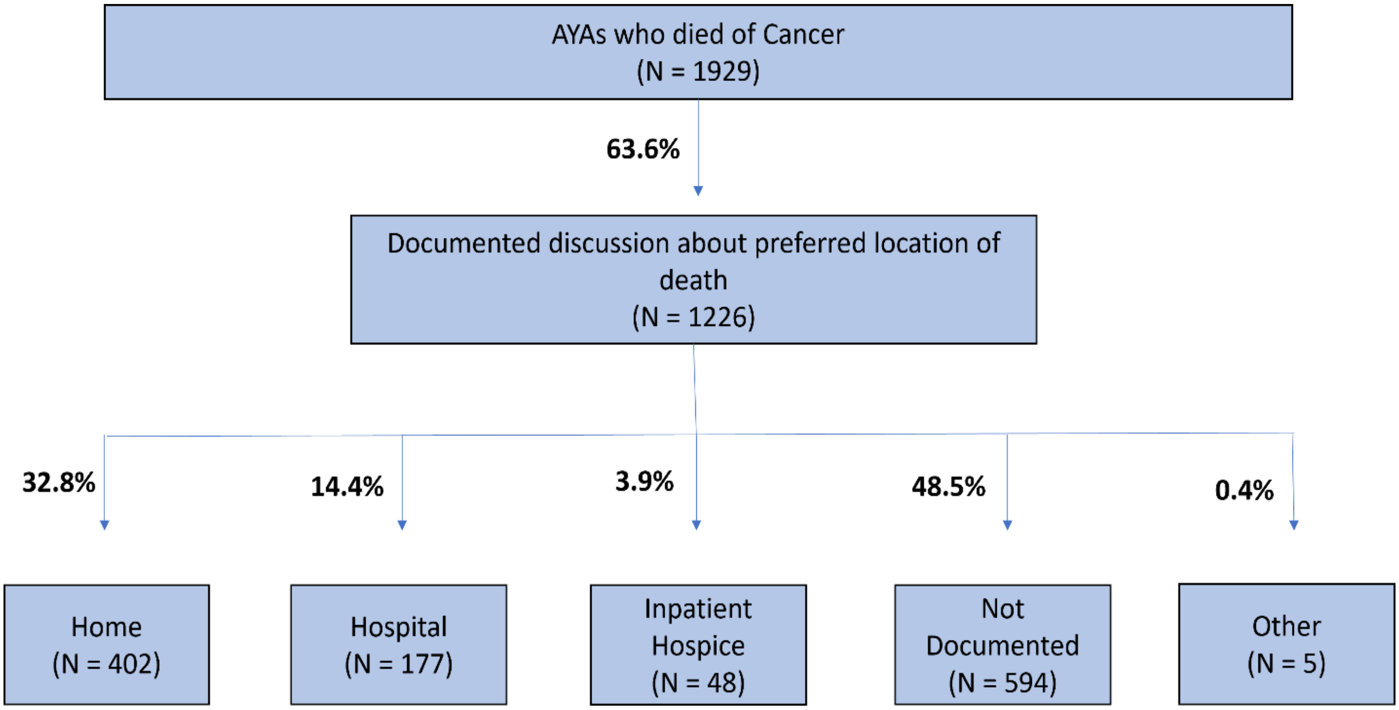

© 2025 Odejide OO et al. *JAMA Network Open*.
