# Supplementary material for: Preferred and Actual Location of Death in Adolescents and Young Adults With Cancer
Source: JAMA Netw Open. 2025 Jan 14;8(1):e2454000. doi: 10.1001/jamanetworkopen.2024.54000 (PMC11733697; doi:10.1001/jamanetworkopen.2024.54000)
Supplement: Supplement 2. — Data Sharing Statement [file jamanetwopen-e2454000-s002.pdf]

Data Sharing Statement

Odejide. Preferred and Actual Location of Death in Adolescents and Young Adults With Cancer. *JAMA Netw Open*. Published January 14, 2025.
doi:10.1001/jamanetworkopen.2024.54000

Data
Data available: Yes
Data types: Deidentified participant data, Data dictionary
How to access data: jennifer_mack@dfci.harvard.edu
When available: With publication

Supporting Documents
Document types: None

Additional Information
Who can access the data: researchers whose proposed use of the data has been approved and who are in compliance with requirements of the DFCI IRB.
Types of analyses: Proposals will be reviewed within the research team, in collaboration with those making requests, to define the scope of analysis.
Mechanisms of data availability: after approval of a proposal or with a signed data access agreement.
